# Bioactive Antimicrobial Peptides from Food Proteins: Perspectives and Challenges for Controlling Foodborne Pathogens

**DOI:** 10.3390/pathogens12030477

**Published:** 2023-03-17

**Authors:** Jessica Audrey Feijó Corrêa, Tiago de Melo Nazareth, Giovanna Fernandes da Rocha, Fernando Bittencourt Luciano

**Affiliations:** 1 Laboratory of Agri-Food Research and Innovation, School of Medicine and Life Sciences, Pontifícia Universidade Católica do Paraná, R. Imaculada Conceição 1155, Curitiba 80215-901, Brazil; 2 Laboratory of Food Chemistry and Toxicology, Faculty of Pharmacy, University of Valencia, Av. Vicent Andrés Estellés s/n, 46100 Burjassot, Spain

**Keywords:** antimicrobial peptides, encrypted peptides, whey protein, protein-rich by-products, lactic acid bacteria

## Abstract

Bioactive peptides (BAPs) derived from food proteins have been extensively studied for their health benefits, majorly exploring their potential use as nutraceuticals and functional food components. These peptides possess a range of beneficial properties, including antihypertensive, antioxidant, immunomodulatory, and antibacterial activities, and are naturally present within dietary protein sequences. To release food-grade antimicrobial peptides (AMPs), enzymatic protein hydrolysis or microbial fermentation, such as with lactic acid bacteria (LAB), can be employed. The activity of AMPs is influenced by various structural characteristics, including the amino acid composition, three-dimensional conformation, liquid charge, putative domains, and resulting hydrophobicity. This review discusses the synthesis of BAPs and AMPs, their potential for controlling foodborne pathogens, their mechanisms of action, and the challenges and prospects faced by the food industry. BAPs can regulate gut microbiota by promoting the growth of beneficial bacteria or by directly inhibiting pathogenic microorganisms. LAB-promoted hydrolysis of dietary proteins occurs naturally in both the matrix and the gastrointestinal tract. However, several obstacles must be overcome before BAPs can replace antimicrobials in food production. These include the high manufacturing costs of current technologies, limited in vivo and matrix data, and the difficulties associated with standardization and commercial-scale production.

## 1. Introduction

BAPs derived from food proteins have been extensively studied and described due to the recognition of their health-promoting properties. Protein hydrolysates are potential sources of BAPs since many are encrypted in the structure of the native/precursor protein. Once released, they can exert beneficial biological effects in the consumer, such as anti-inflammatory, antihypertensive, hypocholesterolemic, hypoglycemic, antioxidative, and antitumoral activities [1,2].

For instance, several BAPs with inhibitory activity towards angiotensin-converting enzymes (ACEs) have been identified in different food matrices, such as coffee [3], cheese [4], and dry-cured ham [1], demonstrating their potential use in the treatment of hypertension. It is noteworthy that recent studies have demonstrated that the cellular receptors for ACE2 are involved in infection by severe acute respiratory syndrome coronavirus 2 (SARS-CoV-2), the pathogen responsible for the global pandemic of COVID-19 (coronavirus disease 2019) [5,6]. Similar immunomodulatory effects may prevent the progression of foodborne diseases, as observed in the peptide Jelleine-I, from honeybee royal jelly, against *Listeria monocytogenes* infection (Lepidoptera as a model system) [7].

Another interesting biological effect observed in the consumption of BAPs is gut microbiome modulation, which is associated with alterations promoted in the balance of reactive oxygen species (ROS) in the gut [8,9,10]. Recent studies have associated gut microbiome modulation promoted by BAPs with preventing and combating neurodegenerative diseases, such as Parkinson’s disease, Alzheimer’s disease, and dementia [11,12]. The intricate mechanisms involved in the neuroprotective and cognitive-enhancing effects are not yet fully elucidated and seem to vary among the BAPs [11]. Either way, it is known that gut microbiome modulation is also an effective approach to mitigating gastrointestinal infections [13,14,15]. The relative abundances of probiotic and commensal bacteria, as well as their diversity, are strongly related to bioprotection against pathogenic bacteria [16].

In summary, such examples illustrate that studies on BAPs derived from food proteins are required in order to explore their full potential in the pharmacological and nutraceutical fields. Although less explored to date, the antimicrobial bioactivity exerted by these molecules has shown that they can be explored in many areas serving different purposes, with promising applications in the agri-food industry as they are potentially food grade [17,18]. The application of AMPs in this industry has been gaining attention due to their high efficacy against pathogenic and contaminant microorganisms without the hazards associated with antibiotic residues from human and animal use [19]. In addition, as consumers become more concerned about the indiscriminate use of chemical compounds, this industry seeks safe and effective alternatives to replace them [20]. For instance, the use of AMPs figures as a promising alternative to antibiotic growth promoters [21], crop protection agents [22], and preservatives in food [23].

From this perspective, this review gathers what is currently known on the use of hydrolysates and BAPs derived from food proteins for controlling foodborne pathogens, and discusses the prospects for and the challenges to implementation of these promising molecules in the agri-food industry.

## 2. Production of BAPs and Hydrolysates

**Figure 1:**
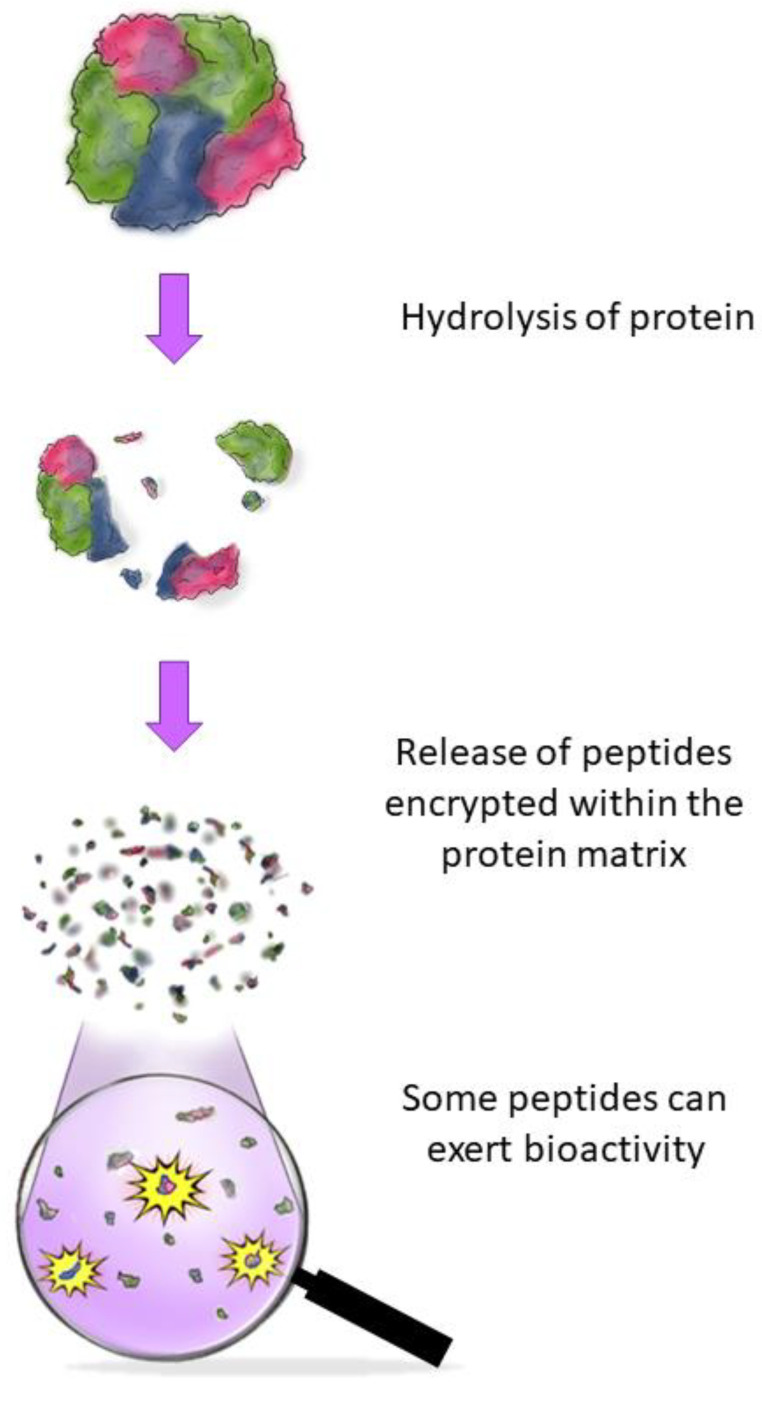
Schematic representation of the generation of BAPs. A generic globular protein is hydrolyzed, releasing the encrypted peptides from the protein matrix. Some of the released peptides may present interesting bioactivities, such as antimicrobial effects towards foodborne pathogens—the focus of this review.

BAPs can be obtained through protein hydrolysis. Figure 1 shows a schematic representation of the generation of BAPs. Food protein hydrolysis is usually done biologically through enzymes or microbial fermentation [24]. For instance, during the gastrointestinal digestion process, BAPs are released from the precursor food protein through intense enzymatic activity—generally involving pepsin, trypsin, and pancreatin [25,26]. Food proteins are also susceptible to hydrolysis during food processing, especially during ripening and fermentation, which can directly influence the profile of generated BAPs. This has been observed in both plant proteins (e.g., in different ripening stages of soybean (*Glycine max* L.) [27]) and animal-origin proteins (e.g., in different cheeses [28,29]).

Although the use of acid and alkaline chemical agents can combine high degrees of protein hydrolysis with low cost, they are not widely used for BAP generation since many alterations in the amino acids occur, presumptively hindering potential bioactivities [24]. Alkaline hydrolysis with hydroxides, for instance, can completely destroy most amino acids and is used for only a few applications in the food industry—mostly restricted to producing foaming agents [30]. On the other hand, many acid protein hydrolysates are used in the food industry as flavor enhancers, although, the process destroys tryptophan and impairs methionine, glutamine, and asparagine [24,31]. Such outcomes might affect the presumed bioactivities of the generated sequences, undermining the employment of chemical hydrolysis methods for BAP production. Notwithstanding, studies on brewer’s-spent-grain hydrolysates (a co-product of the brewing industry) have shown that pre-performing an alkaline protein extraction generally enhances the in vitro bioactivity of enzymatically produced hydrolysates [32].

Anyhow, biological methods are commonly preferred for the GRAS (generally recognized as safe) nature of the generated peptides, as well as for their eco-friendly status [33]. In microbial fermentation, the use of fermentative strains such as LAB is a cost-effective approach [34], considering the additional advantage of the potential removal of hyperallergic and antinutritional factors of the food matrix (e.g., phytate, saponins, and digestive enzyme inhibitors) [24]. It has also been demonstrated that the BAPs generated through microbial fermentation are produced either by protein hydrolysis or by the microorganism itself [35]. However, variations in microbial activity challenge the consistency and reproducibility of microbial hydrolysis processes.

In this sense, hydrolysis of food-sourced proteins by cell-free proteases gains attention, considering that, compared to microbial fermentation, more control can be exerted on the process. In addition, due to its high specificity, favorable environments, and no residual chemicals in the final peptide preparations, enzymatic hydrolysis for BAP production is favored [36]. A wide array of proteases can be used for protein hydrolysis, and these can be obtained from different sources, mainly animals, plants, and microbes [37]. In general, proteases can be classified as exopeptidases or endopeptidases depending on the type of reaction catalyzed by the enzyme. The exopeptidases hydrolyze a peptide bond in the terminal region (N- or C-terminus), as observed for aminopeptidases and carboxypeptidases. As the name suggests, the endopeptidases hydrolyze a peptide bond within an internal region of the protein/peptide. This is the case with pepsin and trypsin, for instance. Within these main classifications, others arise based on the specific targets for cleavage [24].

Another approach worth mentioning is the recombinant expression of BAPs. In the study of Liu and Pan [38], the gene of lunasin—a BAP derived from soybean, which is commercialized as a dietary supplement for health benefits—was synthesized and engineered into *Escherichia coli* for exogenous expression. Moreover, the chemical synthesis of BAPs is also attainable through soluble-phase and solid-phase processes using amino acid units [39]. These are interesting, yet costly, for producing well-characterized and valuable BAPs. In general, the previously described food-derived BAPs with antimicrobial activity were obtained through biological hydrolysis.

Considering further applications, the fractionation and purification processes are required to standardize and concentrate the active molecules from hydrolysates. Membrane filtration is used to recover peptides and amino acids based on specific molecular weight cut-offs. Chromatographic methods are usually employed for purification, including size-exclusion chromatography, ion-exchange chromatography, and reversed-phase high-performance liquid chromatography. In addition, for identification and characterization of BAP sequences, liquid chromatography has usually been combined with mass spectrometry with ionization methods (e.g., electrospray ionization [ESI] and matrix-assisted laser desorption/ionization [MALDI]) [40,41].

### 2.1. Sources of BAPs: A Route to Valorize By-Products

As previously mentioned, the biological activity of the generated peptides varies depending on several factors, such as the processing conditions and the protein source—with the latter being the determinant one. Hypothetically, any protein can generate a BAP through hydrolysis. Considering food proteins, different protein-rich foods, including animal-, plant-, and microbial-sourced (fungal and bacterial), have been associated with BAP production [42,43,44,45].

Notably, many proteins are exclusive of animal organisms, making food of animal origin a valuable source for the generation of specific BAPs. For instance, many of the most-studied BAPs, including lactotransferrin and lactoferricin, are derived from milk [43,46,47]. There has also been a trend of studies into seafood-derived BAPs, especially regarding by-products [34,48,49,50,51].

Exploiting different by-products of the food industry for generating BAPs allows their further valorization. Several studies have addressed the hydrolysis of protein-rich by-products, such as fish processing residues (backbone, skin, head, and abdominal cut-offs), crop and agricultural wastes, waste whey, and low-value meat by-products [52,53,54]. The findings suggest that when peptides are isolated and purified, they have promising applications such as ingredients or preservatives in foods and in the pharmaceutical and nutraceutical fields. They can also be applied as biostimulants or bioprotectors for plants and soil in environmental and agronomic approaches (both hydrolysates and isolated BAPs).

It is worth noting that the microbial fermentation process may also generate bioactive compounds other than peptides, such as organic and phenolic acids, bacteriocins, fatty acids, and peroxides—resulting from the metabolism of the microorganism [55]. The fermentation of by-products has proven itself to be a potential and feasible approach to obtaining BAPs. For instance, in the studies carried out by Ramírez et al. [56,57], spent coffee grounds fermented by *Bacillus clausii* showed an increased abundance of BAPs, and nejayote (maize wastewater) fermented by the same strain exhibited an increased total phenolic content and antioxidant activity. Even though the studies observed these effects separately, both activities are expected to occur considering the capacities of the fermenting bacteria.

Whey, a natural by-product of cheese production, is a frequent subject of study for BAP potential. Fermentation by LAB is usually preferred since they possess great potential to improve the bioactive properties of food combined with the advantages of displaying a high proteolytic activity and being classified as GRAS [58,59]. The topic of LAB will be resumed in Section 4.

Compared to animal protein sources, plant protein sources have more limited potential for the generation of commercial BAPs. Many plants are proven sources of prospective BAPs, in any case. Notable among these are the pulses, which are the dry seeds of annual legumes from the *Fabaceae* (or *Leguminosae*) family [60]. Pulses are a rich protein source, making them potential BAP sources. Seed storage proteins are the most abundant, but pulses also contain proteins considered antinutritional, such as lectins and protease inhibitors, which negatively affect diet quality. However, after hydrolysis, these antinutritional proteins may generate BAPs with potential health-promotion effects (e.g., anticancer activity) [60].

As many pulses, such as soybean and peanuts, are used for edible-oil extraction, there is a massive generation of protein-rich by-products, which can be used for bioactive compound recovery, including BAPs [40]. For instance, high content of proteins of low molecular weight and of peptides can be found in maize nixtamalization wastewater (known as nejayote), which has shown potential anti-inflammatory activity [61].

In short, using protein-rich by-products to generate BAPs is an advantageous and eco-friendly approach to seize and valorize under-utilized and low-cost materials.

### 2.2. Encrypted Peptides and How to Find Them: The Importance of In Silico Approaches

It is possible to investigate the putative antimicrobial activity of peptides in silico with the help of bioinformatics tools [62]. These tools can, for instance, analyze the sequence of food proteins in search of cryptic peptides that acquire this potential when released from the chain that holds them.

Predicting BAPs using in silico approaches is straightforward, as thousands of sequences are able to be screened in much less time than in vitro approaches allow. This, however, must be further combined with experimental assays to prove the predicted bioactivities, as many predictions might not reflect the reality. This was the case in a previous study published by our group [63], in which two whey-derived peptides did not inhibit important bacterial pathogens and mycotoxigenic fungi in vitro despite the prospective in silico predictions.

In silico predictions scan protein databases to identify peptides that match the input criteria. In the study of Brand et al. [64], a methodology for the identification of putative antimicrobial encrypted peptides was proposed based on physiochemical parameters (differential scanning calorimetry and circular dichroism) that may reflect the effects on target-membranes—an important mechanism of action of AMPs (see Section 3.1) [65]. In short, the developed exploratory software Kamal searches within the protein sequence for similarities to known AMPs. This approach complements the traditionally applied prediction methods, which majorly simulate enzymatic cleavages. In those, the action of specific proteases on the protein is simulated to obtain the hydrolysate sequences, which are further investigated for bioactivities based on a database, such as BIOPEP-UWM [66,67,68] or CAMP R3 [69].

The latter, CAMP R3 (Collection of Antimicrobial Peptides), comprises deduced and experimentally proved AMP sequences and also offers prediction tools. Encrypted AMPs were recovered from soybean meal aqueous extract after prediction through analyses using free algorithms provided by CAMP R3 [70]. In the study, hydrolysis was carried out by thermal activation of proteases already present in the extract, and the peptides were recovered through membrane ultrafiltration and chromatographic fractionation.

Apart from discovering encrypted BAPs within a protein sequence, in silico approaches can also be explored to design and optimize them. For instance, in the study by Porto et al. [71], a computational approach was described for the manipulation of a natural AMP from guava (*Psidium guajava* L.). The designed guavanin peptides showed a different mechanism of action to that of most naturally occurring AMPs (i.e., membrane hyperpolarization), which can be further investigated for the development of novel molecules to circumvent the antimicrobial resistance by microorganisms [65].

## 3. The Antimicrobial Activity of Food-Sourced Peptides

The growth in the incidence of foodborne infections poses a risk to the population’s health as well as to the economy. Food contaminated by microorganisms may contain pathogenic bacteria, fungi, parasites, viruses, and toxins, being associated with more than 200 different diseases [23].

Because of this, the use of preservatives is demanded in many different foods to assure safety while maintaining the quality and sensory attributes of the product. In addition, as previously mentioned, natural or minimally-processed antimicrobials are constantly sought to meet consumer trends and minimize the concerns regarding microbial resistance to the synthetic compounds traditionally used as antimicrobial agents [20]. The use of AMPs arises in this scenario.

Among the most used and well-characterized AMPs, the nisins produced by *Lactococcus lactis* subsp. *lactis*, are bacteriocins of the class of lantibiotics widely used as food preservatives. Nisins are classified as GRAS and are a food additive regulated in several countries [72]. In recent studies, nisin was tested as a possible chemotherapeutic for the treatment of bovine mastitis, in which *Staphylococcus* is one of the most important etiological agents, and which often results in prolonged, recurrent, and persistent infections [73,74]. Although not essentially a food-derived AMP, nisin production is often associated with whey valorization processes [75].

Another bioactive molecule studied for the treatment of bovine mastitis is lactoferrin, a protein found in milk. The proteolytic degradation of bovine lactoferrin (naturally occurring during stomach digestion) generates lactoferricin, the most-studied AMP derived from milk. With an amphipathic character and an antiparallel β-sheet structure, lactoferricin and its shorter derivatives display relevant antimicrobial activity. Svendsen et al. [76] suggested that they pose a dual mechanism of action—one related to membrane destabilization and the other to intracellular targets. The general mechanisms of action of AMPs are addressed in Section 3.1.

AMPs have also attracted the attention of the poultry and pork industries due to the growing antimicrobial resistance to conventional antibiotics and the consequent search for effective alternatives for disease control and growth promoters in animal production [77,78]. Studies with weaned piglets showed that feed supplementation with lactoferrin increased the efficiency of weight gain and average daily weight gain [79]. In addition, studies with an artificial peptide (lactoferricin–lactoferrampin fusion) improved growth performance and reduced the occurrence of diarrhea in piglets, with effects similar to those observed with the use of the antibiotic colistin sulfate [80]. These results show the potential use of AMPs as substitutes to antibiotic growth promoters which are usually associated with the rise of antimicrobial resistance worldwide.

Table 1 shows studies associated with using BAPs or hydrolysates from food proteins to control pathogens.

In the context of the AMPs addressed in this review, it should be noted that these molecules can present more than one bioactivity. One important bioactivity is the cytolytic activity against tumor cells, which can also define them as anticancer peptides (ACPs) [93]. Although not the focus of this review, studies with ACPs demonstrate their great chemotherapeutic potential as they generally are specifically toxic to cancer cells. This specificity is due to the existence of electrostatic interactions between cancer cells and ACPs, since these cells generally have a strong negative charge on their cell surface due to the high presence of anionic molecules (e.g., heparin sulfate, and mucins) [93,94]. This negative net charge found in cancer cells is similar to that of prokaryotic membranes, which justifies the dual antimicrobial and anticancer activities of certain peptides. Such mechanisms found for both AMPs and ACPs will be better defined in Section 3.1.

### 3.1. Mechanism of Antimicrobial Activity of BAPs

The knowledge of the molecular mechanisms involved in AMPs activity is essential for their effective application in the agri-food industry. Such information can guide the optimization of the bioactivities through the definition of the promising interactions in a matrix and in vivo (in the case of consumption) and even through genetic engineering of the molecules [95]. However, much remains to be explored and elucidated within this field.

When it comes to size, AMPs are very heterogeneous. Nevertheless, some features are usually shared, including net positive charge and hydrophobicity, which are associated with the ability of AMPs to interact with membrane and/or cytoplasmic components of the microorganisms [65]. Such interaction can occur through nonreceptor-mediated or receptor-mediated mechanisms [96]. In general, most of the known receptor-mediated AMPs are bacteriocins (i.e., produced by bacteria), such as the previously mentioned nisins. Briefly, they act towards a specific target, the receptor, present in the membrane or intracellular component (e.g., DNA and ribosomes), exhibiting a greater specificity compared to nonreceptor-mediated AMPs.

In general, the cell surface of microorganisms presents a net negative charge due to the presence of negatively charged phospholipids and other membrane components, such as teichoic acid (in Gram-positive bacteria) and lipopolysaccharides (in Gram-negative bacteria) [97]. The nonreceptor-mediated mechanism is based on the interaction of AMPs with the membrane, as the peptides generally exhibit a net positive charge. Considering this, the similarity in the activity of AMPs and ACPs is observed, as both prokaryotic cells and cancer cells are preferred targets for the net negative charge they possess. Eukaryotic cells, in contrast, present a more neutral net charge [65,98].

AMP activity is related to different structural determinants, which are based on a first step to the amino acid composition. From this, other important features of antimicrobial activity are defined, such as conformation, charge, domain presence, and hydrophobicity [65].

Focusing on the AMPs, which show a more generalist activity through membrane-targeting, the formation of pores and membrane destabilization, are the outcomes that lead to cell death [99]. Different mechanisms have been proposed for this to occur—AMP insertion into the lipid bilayer to form transmembrane pores and membrane permeabilization through electrostatic interactions—both leading to the collapse of the cell integrity. Additionally, some nonreceptor-mediated AMPs have been described to also present intracellular targets through mechanisms not fully elucidated to date [100]. Anyhow, the translocation of the AMP into the cell is required for this to happen.

#### Gut Microbiota Modulation and Immunomodulation as Mechanisms of Action: Gaps to Be Filled

The gastrointestinal tract comes into contact with several dietary proteins, expressing a wide variety of receptors and regulatory signals as a response to ingested bioactive compounds. Therefore, it has been shown that such modulation of digestive system physiology is essential for the maintenance and improvement of health [101].

The immunomodulatory mechanisms of AMPs, as well as the microbiota modulation they promote in the gut, are closely linked to the benefits to human and animal health that they promote [102].

Immunomodulation occurs when peptides bind to specific receptors in the consumer organism, promoting an immune response and affecting cellular functions. This results in the suppression or stimulation of specific effectors that may enhance the production of antibodies, cytokine expression, lymphocyte activation, or proliferation; and/or non-specific effectors that lead to the activation of macrophages, natural killer cells, and granulocytes [103,104].

AMPs with immunomodulatory activity are essentially produced by organisms with the purpose of acting on their own metabolism. For example, it has been reported in humans that keratinocytes (epidermal cells) produce AMPs that promote the expression of cytokines and chemokines, thus stimulating the immune response to viral infections [105]. However, in food, we ingest many AMPs from the most different sources. The involved immunomodulatory mechanisms are especially important when analyzing the bioactivities and benefits associated with the consumption of certain types of food [106].

As previously mentioned in Section 1, the AMP Jelleine-I affected the membrane integrity, disrupted some intracellular structures, induced the production of ROS, inhibited biofilm formation, and interacted with the DNA of *L. monocytogenes*. In vivo, the administration of Jelleine-I to *Galleria mellonella* infected with the pathogen has shown interesting immunomodulatory effects, including the increase of hemocyte counts, upregulation of other host AMPs, and inhibition of pro-inflammatory cytokines. As a result of the bacterial inhibition, the survival rate of the infected insects increased, and cell proliferation without hemolysis or cytotoxicity was also observed [7].

Beyond regulating pro-inflammatory reactions, AMPs, and BAPs are able to modulate the gut microbiota. The bioprotection of AMPs against pathogens benefits the proliferation of probiotic and commensal bacteria in the gut, which also prevents the colonization of the intestine by pathogenic bacteria. Different mechanisms may be involved in the latter, including competitive exclusion, the consumption of available nutrients, the upregulation of host defense genes, and the production of antimicrobial compounds [7,107,108].

The bioprotection promoted by AMPs towards pathogens is probably related to the disruption of membranes and intracellular targets, mechanisms already described. Additionally, AMPs and BAPs can influence the balance of ROS. In vivo, such influence is believed to occur in the intestine, and is connected to the reduction of oxidative stress. As mentioned in Section 1, such events have been associated with the prevention of neurodegenerative diseases [11,12], as oxidative stress is seen as one of the main villains in the progression of these conditions [109,110,111]. In addition to the repression of ROS generation, many BAPs have antioxidant activity, which may also reduce oxidative stress.

It is worth mentioning that we are considering the AMPs that do not hinder commensal and probiotic bacteria in the gut. This is observed, for instance, in the defensins and cathelicidins, AMPs specialized in host defense that are secreted by the Paneth cells located in the small intestine epithelium [112]. These AMPs are able to inhibit several foodborne pathogens without affecting probiotic and commensal bacteria. Indeed, there are intricate host–microbiota interactions that control the expression of AMPs and suppress pathogen colonization, assuring gut homeostasis [113,114]. This was observed in the study carried out by Cazorla et al. [115], in which microbial probiotics increased the number of Paneth cells and the secretion of AMPs, with consequent enhancement of the antimicrobial activity towards the pathogens *S. aureus* and *Salmonella* Typhimurium in vivo.

In short, there is still much to be elucidated within this field. Further studies will allow the molecular mechanisms involved in the immunomodulatory and gut-microbiota-modulation activities to be understood and explored in the near future.

## 4. The Endless Potential of a Vanguard: Lactic Acid Bacteria

LAB are Gram-positive bacteria responsible for producing a wide variety of bioactive compounds, including hydrogen peroxide, fatty acids, short-chain peptides, and bacteriocins. As many of these compounds exert bioprotective effect against pathogens and deteriorating agents, the significance of LAB in the food industry goes far beyond the production of fermented foods [116,117]. Additionally, these bacteria can adhere to and colonize the digestive system of mammals, exerting probiotic activity [16,118].

It is well known that LAB are important producers of BAPs and AMPs, resulting from the fermentation of products and through the proteolysis of food proteins. LAB strains generally display high proteolytic activity, being able to generate BAPs at a relatively low production cost. Since the proteolytic activity of LAB is strain-dependent, a great variety of proteolytic activities is expected, reflecting an even greater variety of generated BAPs [59,119].

Mostly explored in previous studies are the *Lactococcus*, *Streptococcus*, and *Leuconostoc* genera, as well as the lactobacilli, which are commonly found in foods such as butter, milk, and cheese [120]. The mechanisms of action from which LAB generate BAPs and AMPs are contained in Table 2. Most of what is currently known has come from studies regarding the AMPs produced by LAB through their own metabolic pathways—i.e., bacteriocins. Today, bacteriocins derived from LAB stand out in both commercial and academic spheres [121,122]. Because these bacteria are GRAS, their bacteriocins can be employed as natural preservatives in food. This presents a potential alternative to the synthetic chemicals that have been traditionally used.

However, little is known about the AMPs generated through LAB-promoted hydrolysis of food proteins, even though this is expected to naturally occur in the matrix—considering foods with the presence of LAB—and in the gastrointestinal tract—considering microbiota interactions.

A LAB known to produce AMPs is *Lactiplantibacillus plantarum*, which has been addressed in different studies to assess its potential to inhibit important foodborne pathogens [82,141,142]. Such studies have reported the direct inhibition promoted by *L. plantarum,* cell-free supernatant, and isolated bacteriocins. Additionally, *L. plantarum* has been described to generate AMPs and BAPs through the proteolysis of milk proteins [47,133,143]. BAPs from camel milk fermented with a starter culture of *L. plantarum* were assessed in the study of Muhialdin and Algboory (2018) to determine their benefits to human health, in view of the good health of Iraqi Bedouins who consume the drink regularly. It is believed that fermented camel milk offers intense protection against infections and diseases and can increase the general energy of its consumers [144]. It has also been shown to demonstrate antioxidant [145], antimicrobial [143], antihypertensive, anticancer [146] and antidiabetic [147] activities. The product is used to treat various diseases in Iran, such as jaundice, tuberculosis, anemia, and asthma [144]. Such effects are mainly due to the presence of BAPs derived from milk proteins which are released through the enzymatic proteolysis occurred during fermentation.

Fermented camel milk has shown inhibitory activity against Gram-positive and -negative foodborne pathogenic bacteria, including *E. coli*, *L. monocytogenes*, *S. aureus*, and *Salmonella* Typhimurium [47,148,149]. It has also been demonstrated that the product has a high concentration of AMPs with low molecular weight, and the most active fraction contained 32 AMPs derived from milk proteins [133].

In addition to *L. plantarum* and camel milk, other LAB strains and/or kinds of milk can be used to generate different peptides. For instance, AMPs derived from bovine and goat milk have been described [91,145,150], as well as AMPs obtained through fermentation with *Lactobacillus acidophilus* and *Lactobacillus delbrueckii* subsp. *lactis* [151,152].

## 5. Current Challenges to the Implementation of Bioactive Peptides in the Food Industry

When it comes to pharmaceutical-grade BAPs, the major method of industrial manufacture is chemical synthesis. Despite the high cost and the low output, this is a reliable method for obtaining AMPs for antibiotic substitution, for instance [24,39].

Hydrolysates, BAPs, and AMPs derived from food proteins hold great promise for application as bioactive ingredients or preservatives in the food industry. The preferred method for the release of pharmaceutical- and food-grade BAPs from precursor proteins is through enzymatic hydrolysis [36,153].

In enzymatic hydrolysis, many parameters influence the final product and yield, including the size of the desirable peptides, the employed enzyme, the enzyme-to-substrate ratio, and several physical–chemical conditions, including pressure, temperature, and duration of the process [47].

In addition, the high cost of enzymes, the restricted choice of assured food-grade proteases, and the presence of enzyme inhibitors in the raw content to be hydrolyzed (usually associated with poor yield) remain a challenge for the industry [36].

There has been increasing interest in novel technologies to substitute for or enhance the yield of conventional enzymatic methods for obtaining hydrolysates and BAPs. However, studies are still needed to clarify if the generated BAPs would maintain the specific features and activity, as well as the safety required for application [47]. Anyhow, alternatives to conventional methods are desperately required to ensure a sustainable approach, which is currently lacking, as well as to reduce the high costs.

Regarding the generation of BAPs and AMPs derived from food proteins in the gastrointestinal tract, digestibility and bioavailability are critical aspects to be considered. Although necessary for the liberation of the bioactive molecules from the precursor protein, the action of digestive enzymes may equally lead to the loss of bioactivity of a sequence, depending on where in the sequence the hydrolysis occurs [33]. The peptides that exert their bioactivities in the consumer organism may have resistance to digestion, penetrating through the intestine to reach other tissues. Therefore, simulated digestion assays are essential to predict the bioavailability of BAPs and AMPs after gastrointestinal digestion [154]. However, it is worth noting that in vivo conditions may differ from individual to individual, and such subjectivity hinders standardized application and the formulation of precise regulations [155].

Currently, there is a lack of in vivo studies into BAPs and hydrolysates, especially in human subjects. The studies would provide vital information on their putative interactions with other drugs, for instance. Equally, studies into food matrices are scarce but essential to evaluate the effectiveness and safety of their use as additives or preservatives, considering the possible reactions with food components that might generate undesirable adducts or complexes [19]. Besides, there is an additional factor to be considered when it comes to the use of AMPs—the protective effect of the food matrix in the microorganisms against the action of preservatives [156,157].

Moreover, many of the studies carried out so far give no precise sequences of the BAPs responsible for the antimicrobial activity, which hinders further assays for the determination of their half-maximal inhibitory concentrations (IC_50_) [23,47]. To top it all, little is known about the stability of the molecules during their manufacturing processes [155].

In short, further studies must address such lacunae to allow a future successful implementation of the use of BAPs and AMPs in the food industry.

## 6. Prospects

BAPs are promising molecules for many applications. Many studies have shown their applicability in the pharmacological and nutraceutical fields due to their antioxidant, antidiabetic, antihypertensive, anticancer, anti-inflammatory, hypocholesterolemic, anti-hyperpigmentation, intestine-modulatory, and antimicrobial activities, among others [47,155,158]. To a lesser extent, their potential use in cosmetology and crop improvement has also been described [22,159,160,161,162].

The agri-food industry can benefit from these bioactivities, including, in particular, the antimicrobial potential. The development of safe and efficient alternatives for preservatives and food additives is a constant requirement of this industry. The overuse of antibiotics in animal production is also a major global public health problem due to the emergence of antibiotic resistance. The use of BAPs with antimicrobial activity, i.e., AMPs, figures as an auspicious approach for both food safety and animal-growth-promotion matters [21,163,164].

In addition, recent studies have shown the protective effects of BAPs against the cytotoxicity of the *Fusarium* mycotoxins deoxynivalenol (DON) and T-2 toxin, mainly associated with the decrease of oxidative stress [165,166]. This is also interesting for both food and feed applications since *Fusarium* is a ubiquitous fungal genus that infects crops and produces several types of mycotoxins—which are often found contaminating cereals and cereal-based products [167,168].

Against foodborne pathogens, the use of AMPs derived from food proteins shows great potential, with proven activity against important bacteria, including *L. monocytogenes*, *S. aureus*, *S. enterica*, *E. coli*, *P. aeruginosa*, *K. pneumoniae*, *B. subtilis*, and *V. parahaemolyticus* (Check Table 1 for references). However, most studies have been carried out in vitro, and the in vivo interactions and the interactions with food matrices need to be further evaluated—when considering the use of AMPs as food preservatives or drug. Moreover, the optimal delivery method for food applications must be investigated. This may vary greatly based on the target microorganism, the food, the storage and processing conditions, the evaluated AMP, etc. For instance, the encapsulation of AMPs could be a good approach to enhancing their resistance to undesirable interactions, as well as the formulation of edible films and coatings with AMPs [169].

## 7. Conclusions

In this review, we have provided an overview of what is currently known regarding using BAPs derived from food proteins to control foodborne pathogens.

Despite their great potential, several challenges need to be overcome before the successful implementation of BAPs as antimicrobial alternatives in the food industry. The high production cost of the currently applied technologies, the limited in vivo and matrix data, and the problematic standardization of conditions hinder the industrial scale-up, restricting the potential of AMPs from being fully explored.

## Figures and Tables

**Table 1:** Use of BAPs/hydrolysates derived from food proteins for microbiological control.

BAP/Hydrolysate	Treatment/Evaluation Method	Observed Effects	Reference
Enzymatic hydrolyzed cottonseed meal	In vivo evaluation of supplementation in feed for broiler chicks (15 or 20 g/kg)	Decreased population of *Escherichia coli* and increased *Lactobacilli* counts in ileum; positive effects on zootechnical parameters.	[21]
Enzymatic hydrolyzed cottonseed meal	Challenge in vitro test with *Colletotrichum gloeosporioides, E. coli* O157:H7, and *Staphylococcus aureus.*	Inhibition of *C. gloeosporioides* and *S. aureus* growth; no inhibition on *E. coli* was observed.	[81]
Whey protein hydrolysate	Challenge test with *Listeria monocytogenes*; evaluation in soft cheese-based agar (5 μg/plate), combined or not with LAB strains.	In combination with LAB strains, reduction of *L. monocytogenes* was achieved, although no inhibition was observed when alone.	[82]
Anionic peptide-enriched extract derived from whey proteins	Challenge test with *Listeria innocua* and *L. monocytogenes;* application in reconstituted Cheddar cheese (10 or 20 mg/g) incorporated with lactococci.	Higher anti-listerial activity under higher temperatures and/or low salt content; *L. monocytogenes* more susceptible than *L. innocua;* no inhibition on LAB.	[83]
Encrypted peptides recovered from soybean meal by-product	In vitro evaluation of aqueous extract fractions towards Gram-positive and -negative pathogens; in silico prediction of the antimicrobial sequences.	Inhibition of *S. aureus*, *Acinetobacter* genomospecies, *Aeromonas hydrophila, E. coli, Salmonella enterica,* and *Vibrio parahaemolyticus;* 83 peptide sequences classified as AMP candidates.	[70]
Bovine lactoferrin-derived AMPs	Short (~12 residues-long) AMPs derived from the protein were designed and evaluated towards *Enterococcus faecium* in vivo and ex vivo.	Designed AMPs showed high antimicrobial activity on free cells and biofilm, low mammalian cytotoxicity, and membrane-activating mechanisms.	[84]
Hen egg-white lysozyme-derived AMPs (enzymatic hydrolysis)	Evaluation through a radial diffusion assay.	Antibacterial activity against *Leuconostoc mesenteroides* and *E. coli*, the latter showing greater susceptibility.	[85]
Egg albumin hydrolysates (enzymatic hydrolysis)	Evaluation of the antimicrobial activity of hydrolysates through the disc diffusion and tube dilution method.	Antibacterial activity against *L. monocytogenes*, *Bacillus cereus*, *S. aureus*, *Salmonella* Typhimurium, *Streptococcus pyogenes, Klebsiella oxytoca*, *Pseudomonas aeruginosa*, *Bacillus subtilis*, *Listeria ivanovii,* and *E. coli*.	[86]
Goat- and bovine-milk-derived BAPs (enzymatic hydrolysis)	After fungal proteolysis, evaluation through disk diffusion towards bacterial and fungal microorganisms.	Antimicrobial activity towards *L. monocytogenes*, *S. aureus, E. coli*, *S. enterica*, *P. aeruginosa, Fusarium oxysporum, Penicillium expansum,* and *Candida albicans*; no inhibition against *Aspergillus fumigatus* was observed.	[87]
Water-soluble AMPs recovered from the ripened Brazilian Canastra artisanal Minas cheese	Evaluation of the promoted inhibition on *E. coli,* comparing different ripening stages and cheese producers; identification of peptide sequences.	Observed variations influenced by temperature, pH, and other manufacturing characteristics; identification of six validated AMPs, 8–14 residues long, derived from caseins.	[88]
Peptide-rich fractions extracted from Spanish dry-cured ham	128 fractions chromatographically purified were evaluated through agar-well-diffusion assay for the inhibition of *L. monocytogenes* and *L. innocua;* peptidomic study on the naturally generated BAPs.	Two fractions showed inhibitory effects towards *Listeria* strains; identification of 105 BAPs in the two bioactive fractions, 10 with anti-listerial activity.	[89]
Bovine collagen hydrolysates (enzymatic hydrolysis)	Antimicrobial activity was evaluated for the hydrolysates (0.5 to 5 mg/mL); peptide profiling of hydrolysates.	Hydrolysates showed inhibitory activity towards *E. coli, S. aureus*, and *B. subtilis;* no inhibition was achieved against *E. faecalis, P. aeruginosa,* or *Klebsiella pneumoniae.* Identification of several peptides with low molar mass (<2 kDa).	[90]
Goat-whey hydrolysates (enzymatic hydrolysis)	Evaluation of the antimicrobial activity of hydrolysates through disc diffusion method; peptide profiling of fractions.	The hydrolysate showed bactericidal effects towards *B. cereus, Salmonella* Typhimurium, and *E. coli;* and bacteriostatic activity against *S. aureus.* Two peptides accounted for the bioactivity.	[91]
Rainbow trout by-product hydrolysates (enzymatic hydrolysis)	Assessment of inhibitory activity against several bacterial strains.	Inhibitory activity was detected towards all tested strains, with the highest activity against *Flavobacterium* species; prolonged lag phase of bacterial growth.	[92]

**Table 2:** Lactic acid bacteria and their mechanisms to produce AMPs.

Genus/Species	Studied Food Product	Mechanism of AMP Production	References
***Lactococcus***			
*Lactococcus lactis lactis*	Skim milk	Ribosomal synthesis of bacteriocins	[123,124]
*Lactococcus lactis lactis*	Cottage cheese	Protein hydrolysis, releasing BAPs	[125]
***Streptococcus***			
*Streptococcus thermophilus*	Milk, yogurt, soft and hard cheeses	Ribosomal synthesis of bacteriocins	[126,127,128]
***Lactobacillus***			
*Lactobacillus acidophilus*	Cheese, yogurt	Protein hydrolysis, releasing free amino acids and BAPs	[119,129]
*Lactobacillus gasseri*	Yogurt	Protein hydrolysis, releasing free amino acids and BAPs	[119,130]
*Lactobacillus helveticus*	Skim milk supplemented with whey protein	Protein hydrolysis, releasing free BAPs	[131]
*Lactobacillus delbrueckii bulgaricus*	Skimmed goat milk	Protein hydrolysis, releasing free amino acids and BAPs	[132]
***Lactiplantibacillus***			
*Lactiplantibacillus plantarum*	Fermented camel milk	Protein hydrolysis, releasing free amino acids and BAPs	[119,133]
*Lactiplantibacillus plantarum*	Pineapple	Protein hydrolysis, releasing free amino acids and BAPs	[134]
*Lactiplantibacillus plantarum*	Wheat grain	Protein hydrolysis, releasing free amino acids and BAPs	[135]
***Leuconostoc***			
*Leuconostoc mesenteroides cremoris*	Cheese, butter, heavy cream	Ribosomal synthesis of bacteriocins	[136,137,138]
***Pediococcus***			
*Pediococcus pentosaceus*	Fermented pork sausage	Production of pediocin PA-1/AcH by protein hydrolysis	[139]
***Enterococcus***			
*Enterococcus faecium*	Minas cheese	Ribosomal synthesis of bacteriocins	[140]
*Enterococcus mundtii*	Minas cheese	Ribosomal synthesis of bacteriocins	[140]

## Data Availability

Not applicable.
